# Survival benefit of neoadjuvant chemotherapy in HR+/HER2− early breast cancer stratified by luminal B status and clinical risk: a real-world cohort study in a Chinese population

**DOI:** 10.3389/fonc.2026.1804093

**Published:** 2026-04-13

**Authors:** Wei Wang, Xueyan Liang, Yuchao Yang, Wenhai Zhang, Denghua Huang, Jiajun Li, Yukun Liu, Hui Ren

**Affiliations:** 1 Second Department of Oncology Radiotherapy, Qingdao Central Hospital, University of Health and Rehabilitation Sciences, Qingdao, Shandong, China; 2 Department of Breast Surgery, Qingdao Central Hospital, University of Health and Rehabilitation Sciences, Qingdao, Shandong, China; 3 Department of Breast Surgery, Eighth Affiliated Hospital of Guangxi Medical University, Guigang City People’s Hospital, Guigang, Guangxi, China

**Keywords:** breast neoplasms, Luminal B, neoadjuvant chemotherapy, real-world study, survival

## Abstract

**Background:**

The role of neoadjuvant chemotherapy (NACT) in hormone receptor-positive/HER2-negative (HR+/HER2−) breast cancer remains controversial. This real-world study evaluated the impact of NACT on overall survival (OS) and disease-free survival (DFS), specifically focusing on differential benefits within Luminal B and high-risk Luminal B subgroups.

**Methods:**

We retrospectively analyzed 990 patients with HR+/HER2− invasive breast cancer treated between 2013 and 2022. Patients received either NACT (n=195) or upfront surgery (n=795). “High-risk Luminal B” was defined as Luminal B subtype combined with clinical T3, N2, or Ki-67 ≥30%. Multivariable Cox regression adjusted for confounders to assess prognostic factors. Interaction tests evaluated heterogeneity in treatment effects across subgroups.

**Results:**

Despite higher baseline risk in the NACT group (larger tumors, higher grade), NACT was independently associated with better OS (HR 0.60, 95% CI 0.37–0.99) and DFS (HR 0.61, 95% CI 0.42–0.90) in the overall population. This benefit was most pronounced in the Luminal B subgroup (OS HR 0.47, 95% CI 0.26–0.84). A significant interaction was observed between treatment and risk status for OS (P for interaction = 0.026). Specifically, high-risk Luminal B patients achieved significantly higher 5-year OS with NACT (91.5% vs. 80.9%), whereas non-high-risk patients derived no survival benefit (adjusted HR 1.30). DFS showed consistent numerical trends but lacked significant interaction.

**Conclusion:**

NACT provided a survival advantage in this real-world HR+/HER2− cohort, with benefits primarily concentrated in high-risk Luminal B patients characterized by aggressive biology and high tumor burden. These findings support a risk-adapted strategy prioritizing NACT for this high-risk population while suggesting caution for lower-risk subgroups.

## Introduction

Breast cancer is a biologically heterogeneous disease that is routinely classified into clinically relevant subgroups according to the expression of estrogen receptor (ER), progesterone receptor (PR), and human epidermal growth factor receptor 2 (HER2) (1). The expression status of these molecular markers is also widely used to guide therapeutic decision-making and to evaluate prognosis in breast cancer patients (2). Hormone receptor-positive, human epidermal growth factor receptor 2-negative (HR+/HER2−) breast cancer accounts for the largest proportion of breast cancer cases globally and generally has a more favorable prognosis compared with triple-negative and HER2-positive subtypes (3). However, substantial biological heterogeneity exists within this population (4–6). Specifically, Luminal B-like tumors are often characterized by higher proliferative activity, higher histological grade, and a risk of earlier recurrence (7–9). Despite their sensitivity to endocrine therapy, these tumors contribute significantly to breast cancer-related mortality. A critical challenge in current clinical practice remains the precise identification of biologically high-risk patients within the HR+/HER2− population who truly require intensified perioperative systemic therapy.

Neoadjuvant chemotherapy (NACT), defined as systemic chemotherapy administered before definitive surgery, has been established as the standard of care for triple-negative and HER2-positive breast cancers. It significantly improves pathological complete response (pCR) rates, increases opportunities for breast-conserving surgery, and informs the escalation of adjuvant therapy based on residual disease (10–12). In contrast, the role of NACT in HR+/HER2− breast cancer is far less defined. Previous clinical trials and real-world studies have consistently shown that HR+/HER2− tumors exhibit lower chemosensitivity and markedly lower pCR rates (13–15). Furthermore, pCR has limited value as a surrogate for prognosis in this subtype (16, 17). Consequently, domestic and international guidelines generally regard HR+/HER2− status as a “selective indication” for NACT, reserving it primarily for locally advanced cases requiring downstaging to facilitate operability or breast conservation. Meanwhile, a substantial proportion of patients with Luminal-like disease continue to be managed with upfront surgery followed by adjuvant chemotherapy and endocrine therapy, or, in some cases, neoadjuvant endocrine therapy.

Current research on NACT in the HR+/HER2− population has several limitations. First, many studies have focused primarily on short-term endpoints such as pCR, breast conservation rates, or axillary downstaging (18), lacking systematic evaluations of long-term outcomes like overall survival (OS) and disease-free survival (DFS) (19). Second, HR+/HER2− breast cancer is often analyzed as a single entity, with insufficient attention paid to the biological distinctions between Luminal A and Luminal B subtypes, or to the combined risk profile of “biological high risk plus clinical high risk” (20, 21). Third, studies directly comparing NACT versus upfront surgery outcomes within the same HR+/HER2− population are limited and often fail to adequately adjust for baseline imbalances in T/N stage, grade, and proliferation indices, making it difficult to assess the true impact of NACT on long-term prognosis (22). As a result, high-quality evidence is still lacking regarding whether NACT truly improves long-term outcomes for HR+/HER2− patients and whether any benefit is concentrated in specific subgroups.

Of particular interest is the so-called “high-risk Luminal B” population—patients who combine Luminal B biology with higher T/N stages and significantly elevated Ki-67 indices (23, 24). This group possesses both biological aggressiveness and high clinical risk features, theoretically making them more likely to benefit from intensified chemotherapy, including NACT. However, specific studies targeting this subgroup are scarce, and systematic evaluations of their OS and DFS benefits following NACT in real-world settings, as well as their relative advantage compared with other HR+/HER2− patients, are lacking.

In this context, we conducted a single-center, large-scale, real-world retrospective cohort study to systematically compare the impact of NACT versus upfront surgery on OS and DFS in early HR+/HER2− breast cancer. We utilized standardized immunohistochemical surrogate criteria to stratify Luminal subtypes (25–27) and prespecified a “high-risk Luminal B” subgroup, reclassifying risk by integrating biological markers with clinical staging. Multivariable Cox regression, subgroup analyses, and interaction tests were performed in the overall population, the Luminal B subgroup, and the high-risk Luminal B subgroup. This study aimed to answer three core questions: (1) Is NACT associated with improved long-term prognosis in a real-world HR+/HER2− population? (2) Is this potential benefit more pronounced in Luminal B patients? (3) Do high-risk Luminal B patients derive a greater relative benefit compared with other subgroups? Our findings aim to provide evidence for the risk-adapted application of NACT in the HR+/HER2− population and to promote the individualization of perioperative treatment strategies.

## Materials and methods

### Study design and ethics

This was a single-center, retrospective cohort study. The study protocol was approved by the Ethics Committee of Guigang City People’s Hospital (Approval No.: GYLLPJ-20220121-09). The requirement for informed consent was waived by the Ethics Committee due to the retrospective nature of the study and the use of anonymized historical data. The study was conducted in accordance with the Declaration of Helsinki and relevant regulations.

### Study population and outcomes

We screened patients with invasive breast cancer treated at Guigang City People’s Hospital between 2013 and 2022. After pathological diagnosis, treatment was initiated according to routine institutional clinical practice following standard pre-treatment evaluation. The inclusion criteria were: (1) female gender, age ≥18 years; (2) pathologically confirmed invasive breast cancer; (3) HR-positive (ER or PR ≥1%) and HER2-negative status; (4) availability of complete baseline clinicopathologic information; and (5) availability of survival data for follow-up.

The exclusion criteria were: (1) presence of distant metastasis at initial diagnosis (Stage IV); (2) inflammatory breast cancer; (3) history of other malignancies within the past 5 years; (4) missing information regarding neoadjuvant chemotherapy (NACT); or (5) missing outcome data for overall survival (OS) or disease-free survival (DFS).

Patients in the NACT group received neoadjuvant regimens based on anthracyclines and/or taxanes. All patients underwent radical surgical treatment and received adjuvant systemic therapy according to standard guidelines. All HR-positive patients received endocrine therapy. Clinical data, pathological information, treatment details, and follow-up outcomes were extracted from the electronic medical record system.

Ultimately, 990 patients were included in the analysis, of whom 195 (19.7%) received NACT and 795 (80.3%) underwent upfront surgery.

### Study endpoints

Overall Survival (OS): Defined as the time from diagnosis to death from any cause.

Disease-Free Survival (DFS): Defined as the time from diagnosis to the first occurrence of any of the following events: (1) ipsilateral invasive breast cancer recurrence; (2) regional lymph node recurrence; (3) distant metastasis; (4) contralateral invasive breast cancer; or (5) death from any cause.

### Clinicopathologic variables and definitions

Baseline variables recorded included age, menopausal status, tumor size, clinical T/N stage, histological grade, ER/PR expression percentages, Ki-67 index, and molecular subtype.

### Pathological assessment

Tumor specimens were evaluated by experienced breast pathologists according to standard pathological procedures at Guigang City People’s Hospital. Expression of estrogen receptor (ER), progesterone receptor (PR), human epidermal growth factor receptor 2 (HER2), and Ki-67 was assessed using immunohistochemistry (IHC) on formalin-fixed paraffin-embedded tumor tissue. ER and PR positivity were defined as nuclear staining in ≥1% of tumor cells according to established international guidelines. HER2 status was determined according to the American Society of Clinical Oncology/College of American Pathologists (ASCO/CAP) guidelines. Cases with equivocal HER2 IHC results were further evaluated by *in situ* hybridization when necessary. The Ki-67 proliferation index was evaluated using immunohistochemical staining and recorded as the percentage of positively stained tumor cell nuclei in representative tumor areas, as described in previous pathological studies (28).

All clinicopathologic variables, including patient demographics, tumor characteristics (tumor size, T stage, N stage, histological grade), biomarker expression (ER, PR, HER2, Ki-67), treatment information, and follow-up outcomes, were extracted from the institutional electronic medical record database. The database was established from routinely collected clinical records and maintained by the hospital information system.

### Neoadjuvant chemotherapy regimens

Patients in the NACT group received neoadjuvant chemotherapy based on anthracycline- and/or taxane-containing regimens according to institutional practice and contemporary treatment guidelines. Common regimens included epirubicin plus cyclophosphamide followed by a taxane (EC-T) or docetaxel-based combinations. Epirubicin was typically administered at a dose of 90 mg/m² and cyclophosphamide at 600 mg/m² every 3 weeks. Taxanes, such as paclitaxel (80 mg/m² weekly) or docetaxel (75–100 mg/m² every 3 weeks), were administered according to standard clinical protocols. Neoadjuvant chemotherapy was generally delivered for 4–8 cycles, depending on the regimen and clinical response. Treatment regimens and the number of cycles were determined by treating oncologists according to tumor characteristics, patient tolerance, and prevailing clinical guidelines during the study period.

### Definition of luminal B subtype

Based on the immunohistochemical surrogate definitions from the St. Gallen consensus, HR-positive/HER2-negative tumors were further classified into Luminal A-like and Luminal B-like subtypes. Luminal B-like (HER2-negative) was defined as ER-positive and HER2-negative, combined with at least one of the following high-risk features: Ki-67 ≥20%, PR <20%, or histological grade 3. This operational definition was derived from the 2013 St. Gallen consensus and served as the standard classification method at our center. It aligns with recent St. Gallen guidelines recommending the use of Ki-67, grade, and PR levels to characterize the Luminal B biological phenotype.

### Definition of high-risk luminal B subgroup

The “High-Risk Luminal B” subgroup was defined as meeting the standard Luminal B criteria plus at least one of the following high-risk features: (1) clinical T3 stage; (2) clinical N2 stage; or (3) Ki-67 index ≥30%. This definition was intended to capture patients with both biologically aggressive tumor characteristics and high clinical tumor burden, representing a clinically meaningful high-risk subgroup within the HR+/HER2− population.

### Statistical analysis

All statistical analyses were performed using R software (version 4.5.1; R Foundation for Statistical Computing, Vienna, Austria), utilizing packages including survival, survminer, dplyr, and forest plot. Continuous variables are presented as mean ± standard deviation (SD) for normally distributed data or median with interquartile range (IQR) for skewed data. Categorical variables are expressed as frequencies and percentages [n (%)]. Differences between groups were assessed using the Student’s t-test or Wilcoxon rank-sum test for continuous variables, and the Chi-square test or Fisher’s exact test for categorical variables, as appropriate.

Survival curves for OS and DFS were generated using the Kaplan–Meier method, and differences between treatment groups were compared using the log-rank test. Median follow-up time and survival rates were calculated and reported according to standard methods. Univariable Cox proportional hazards models were constructed to identify potential prognostic factors for OS and DFS. Variables that were clinically relevant or had a P-value <0.10 in univariable analysis were included in the multivariable Cox model. Covariates included age, menopausal status, T stage, N stage, tumor size, histological grade, ER expression (%), PR expression (%), Ki-67 index (%), molecular subtype (Luminal A vs. B), and receipt of NACT. Results are presented as hazard ratios (HRs) with 95% confidence intervals (CIs).

To evaluate the treatment effect of NACT on OS and DFS across different clinical contexts, subgroup analyses were performed in prespecified clinically relevant subgroups (stratified by age, menopausal status, T/N stage, histological grade, Luminal subtype, and high-risk Luminal B status). Heterogeneity of the treatment effect across subgroups was assessed by including an interaction term (“treatment × subgroup variable”) in the Cox models. A two-sided P-value <0.05 for the interaction term was considered indicative of statistically significant heterogeneity.

All statistical tests were two-sided, and a P-value <0.05 was considered statistically significant.

## Results

### Baseline characteristics of the study population

The baseline clinicopathologic characteristics of the overall study population are summarized in **Table 1**. A total of 990 patients were included, of whom 195 (19.7%) received neoadjuvant chemotherapy (NACT) and 795 (80.3%) underwent upfront surgery. The mean age was similar between the two groups (NACT: 51 ± 9 years vs. Non-NACT: 52 ± 9 years; P = 0.386).

**Table 1:** Baseline clinicopathologic characteristics of patients receiving neoadjuvant chemotherapy versus upfront surgery.

Characteristic	Neoadjuvant chemotherapy		p-value
	No (N = 795)	Yes(N = 195)	
Age, Mean ± SD	52 ± 9	51 ± 9	0.3861
Tumor size (cm), Mean ± SD	2.91 ± 1.56	3.27 ± 1.74	0.0101
Postmenopausal, n (%)			0.8732
No	370 (46.5%)	92 (47.2%)	
Yes	425 (53.5%)	103 (52.8%)	
T stage, n (%)			0.0192
T1	328 (41.3%)	68 (34.9%)	
T2	328 (41.3%)	76 (39.0%)	
T3	139 (17.5%)	51 (26.2%)	
N stage, n (%)			0.3692
N0	398 (50.1%)	93 (47.7%)	
N1	297 (37.4%)	70 (35.9%)	
N2	100 (12.6%)	32 (16.4%)	
Grade, n (%)			0.0012
1	206 (25.9%)	43 (22.1%)	
2	383 (48.2%)	76 (39.0%)	
3	206 (25.9%)	76 (39.0%)	
Luminal subtype, n (%)			0.0472
Luminal A	214 (26.9%)	39 (20.0%)	
Luminal B	581 (73.1%)	156 (80.0%)	
ER percent, Median (Q1, Q3)	85 (78, 92)	87 (81, 93)	0.0041
PR percent, Median (Q1, Q3)	70 (56, 82)	68 (54, 81)	0.2531
Ki67 percent, Median (Q1, Q3)	29 (19, 40)	33 (24, 41)	0.0121

Values are presented as mean ± standard deviation (SD), median with interquartile range (IQR), or number (percentage), as appropriate.

P values were calculated using the Student’s t-test for continuous variables with normal distribution, the Wilcoxon rank-sum test for non-normal continuous variables, and the chi-square test for categorical variables.

ER, estrogen receptor; PR, progesterone receptor.

Compared with patients who did not receive NACT, those in the NACT group had larger tumor diameters (3.27 ± 1.74 cm vs. 2.91 ± 1.56 cm; P = 0.010). Menopausal status did not differ significantly between the groups (P = 0.873). Regarding clinical T stage, T3 tumors were more common in the NACT group (26.2% vs. 17.5%), whereas T1 tumors were more frequent in the non-NACT group (34.9% vs. 41.3%), with a statistically significant difference (P = 0.019). No significant difference was observed in clinical N stage (P = 0.369).

Significant differences were found in histological grade (P = 0.001), with a higher proportion of Grade 3 tumors in the NACT group (39.0% vs. 25.9%). Similarly, the distribution of molecular subtypes differed (P = 0.047), with a higher prevalence of the Luminal B subtype in the NACT group (80.0% vs. 73.1%).

Regarding hormone receptor expression, median ER expression was slightly higher in the NACT group (87% [IQR 81–93] vs. 85% [IQR 78–92]; P = 0.004), while PR expression showed no significant difference (P = 0.253). Ki-67 expression was significantly higher in the NACT group (33% [IQR 24–41] vs. 29% [IQR 19–40]; P = 0.012), consistent with the higher proportion of high-grade and Luminal B tumors in this group.

### Prognostic factors for overall survival and disease-free survival in the overall population

**Figure 1:**
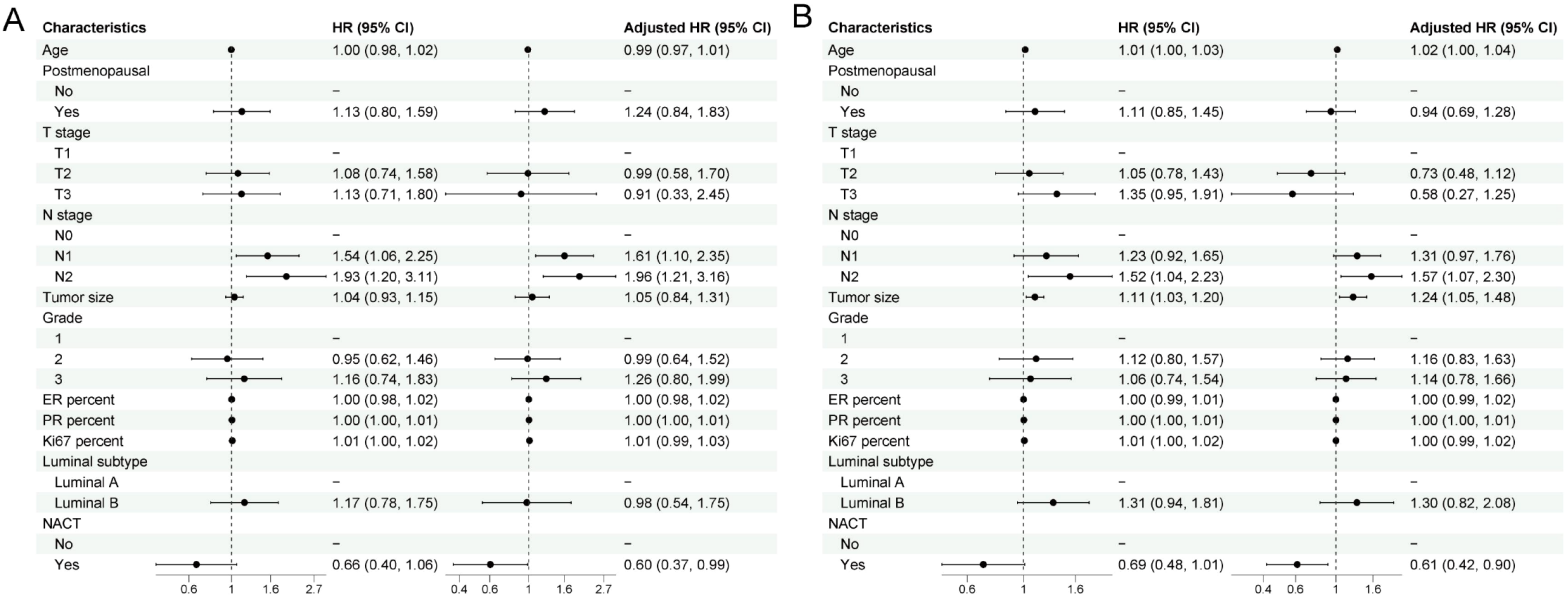
Univariable and multivariable Cox regression analyses of overall survival **(A)** and disease–free survival **(B)** in the overall population. Forest plots display hazard ratios (HRs) and 95% confidence intervals (CIs) for clinicopathologic characteristics associated with overall survival (OS) **(A)** and disease–free survival (DFS) **(B)** in the overall cohort. Left columns show univariable Cox models, and right columns show multivariable Cox models adjusted for age, menopausal status, T stage, N stage, tumor size, histologic grade, ER percentage, PR percentage, Ki-67 index, luminal subtype, and receipt of neoadjuvant chemotherapy (NACT). The dashed vertical line represents HR = 1.0. HRs <1 indicate lower risk, and HRs >1 indicate higher risk for the corresponding outcome.

The median follow-up time, estimated using the reverse Kaplan–Meier method, was 61.0 months. Univariable and multivariable Cox regression analyses for overall survival (OS) and disease-free survival (DFS) in the overall population are presented in **Figure 1**.

In the OS analysis (**Figure 1A**), higher clinical N stage was significantly associated with worse survival. Compared with N0 stage, the multivariable-adjusted hazard ratios (HRs) were 1.61 (95% CI 1.10–2.35) for N1 and 1.96 (95% CI 1.21–3.16) for N2. Histological grade and biological markers such as ER%, PR%, and Ki-67 did not show independent prognostic significance in the multivariable analysis. Patients who received NACT had a lower risk of death, with an adjusted HR of 0.60 (95% CI 0.37–0.99).

In the DFS analysis (**Figure 1B**), N stage remained the primary adverse prognostic factor, with adjusted HRs of 1.31 (95% CI 0.97–1.76) for N1 and 1.57 (95% CI 1.07–2.30) for N2. Tumor size showed a borderline association in the multivariable model. Patients receiving NACT continued to demonstrate a lower risk of recurrence in terms of DFS, with an adjusted HR of 0.61 (95% CI 0.42–0.90).

### Prognostic factors for overall survival and disease-free survival in the luminal B subgroup

**Figure 2:**
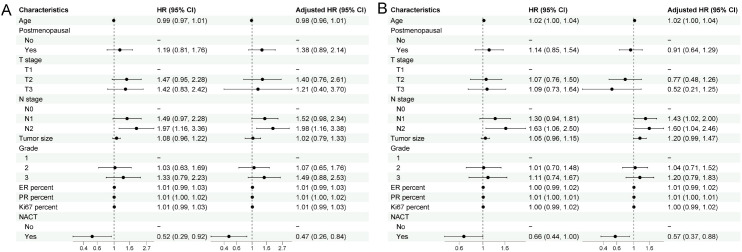
Univariable and multivariable Cox regression analyses of overall survival **(A)** and disease–free survival **(B)** in the Luminal B subgroup. Forest plots show hazard ratios (HRs) and 95% confidence intervals (CIs) for clinicopathologic characteristics associated with overall survival (OS) **(A)** and disease–free survival (DFS) **(B)** in the Luminal B subgroup. Left panels display univariable Cox models, and right panels display multivariable Cox models adjusted for age, menopausal status, T stage, N stage, tumor size, histologic grade, ER percentage, PR percentage, Ki-67 index, and receipt of neoadjuvant chemotherapy (NACT). The dashed vertical line indicates HR = 1.0. HRs <1 represent decreased risk, whereas HRs >1 represent increased risk for the respective outcomes.

Univariable and multivariable Cox regression analyses for OS and DFS in the Luminal B subgroup are shown in **Figure 2**.

In the OS analysis (**Figure 2A**), clinical N stage remained the strongest prognostic factor. Compared with N0, the adjusted HR was 1.52 (95% CI 0.98–2.34) for N1 and 1.98 (95% CI 1.16–3.38) for N2. Other clinicopathologic factors—including tumor size, histological grade, ER%, PR%, and Ki-67%—did not demonstrate independent statistical significance in the multivariable model. NACT was associated with a lower risk of death, with an adjusted HR of 0.47 (95% CI 0.26–0.84).

In the DFS analysis (**Figure 2B**), the prognostic pattern was similar to that of OS, with higher N stage independently associated with worse outcomes. The adjusted HRs were 1.43 (95% CI 1.02–2.00) for N1 and 1.60 (95% CI 1.04–2.46) for N2. Tumor size showed a borderline association (HR 1.20; 95% CI 0.99–1.47). NACT remained associated with better DFS, with an adjusted HR of 0.57 (95% CI 0.37–0.88).

### Survival outcomes according to neoadjuvant chemotherapy in the overall cohort and luminal B subgroups

OS and DFS rates across different populations are presented in **Table 2**. In the overall population, survival rates were generally better in the NACT group compared with the non-NACT group. The 5-year OS was 89.5% in the NACT group versus 84.1% in the non-NACT group. A similar trend was observed in the Luminal B subgroup: the 5-year OS was 91.1% in the NACT group, higher than 83.4% in the non-NACT group. This difference was most pronounced in the prespecified high-risk Luminal B subgroup, where the 5-year OS reached 91.5% in the NACT group compared with only 80.9% in the non-NACT group.

**Table 2:** One-, three-, and five-year OS and DFS according to NACT status in the overall cohort, Luminal B subgroup, and high-risk Luminal B subgroup.

Population	Endpoint	NACT group	1-year survival (%)	3-year survival (%)	5-year survival (%)
Overall population	OS	No NACT	100	92.6	84.1
		NACT	100	94.8	89.5
	DFS	No NACT	100	97.4	84.5
		NACT	100	97.3	88.8
Luminal B subgroup	OS	No NACT	100	92.2	83.4
		NACT	100	95.4	91.1
	DFS	No NACT	100	97.7	84.8
		NACT	100	97.3	89
High-risk Luminal B subgroup	OS	No NACT	100	91.1	80.9
		NACT	100	95.1	91.5
	DFS	No NACT	100	97.5	83.3
		NACT	100	96.6	88.2

Survival probabilities were estimated using Kaplan–Meier analysis. Percentages represent survival probability at the corresponding time points. NACT, neoadjuvant chemotherapy.

DFS rates showed a consistent trend. In the overall population, Luminal B subgroup, and high-risk Luminal B subgroup, the NACT group consistently displayed numerically higher 5-year DFS rates (88.8% vs. 84.5%, 89.0% vs. 84.8%, and 88.2% vs. 83.3%, respectively). Although some comparisons did not reach statistical significance, the overall direction consistently supported a potential benefit of NACT.

**Figure 3:**
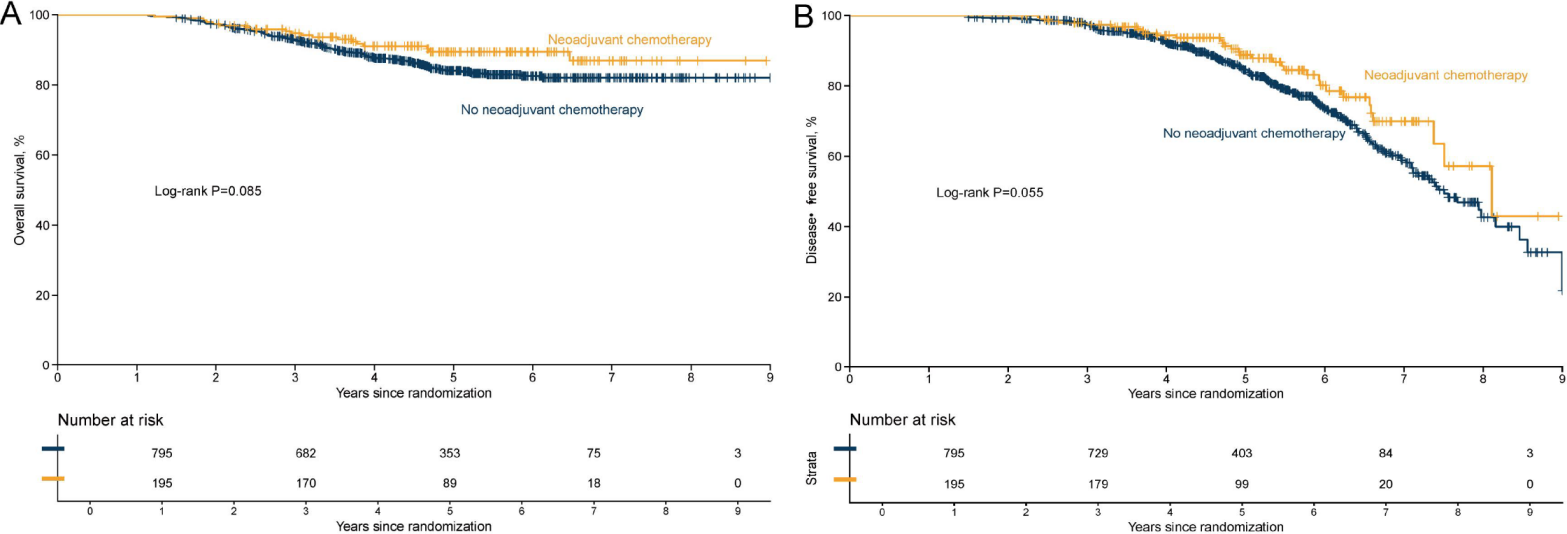
Kaplan–Meier curves of overall survival **(A)** and disease-free survival **(B)** in the overall population by neoadjuvant chemotherapy status.

**Figure 4:**
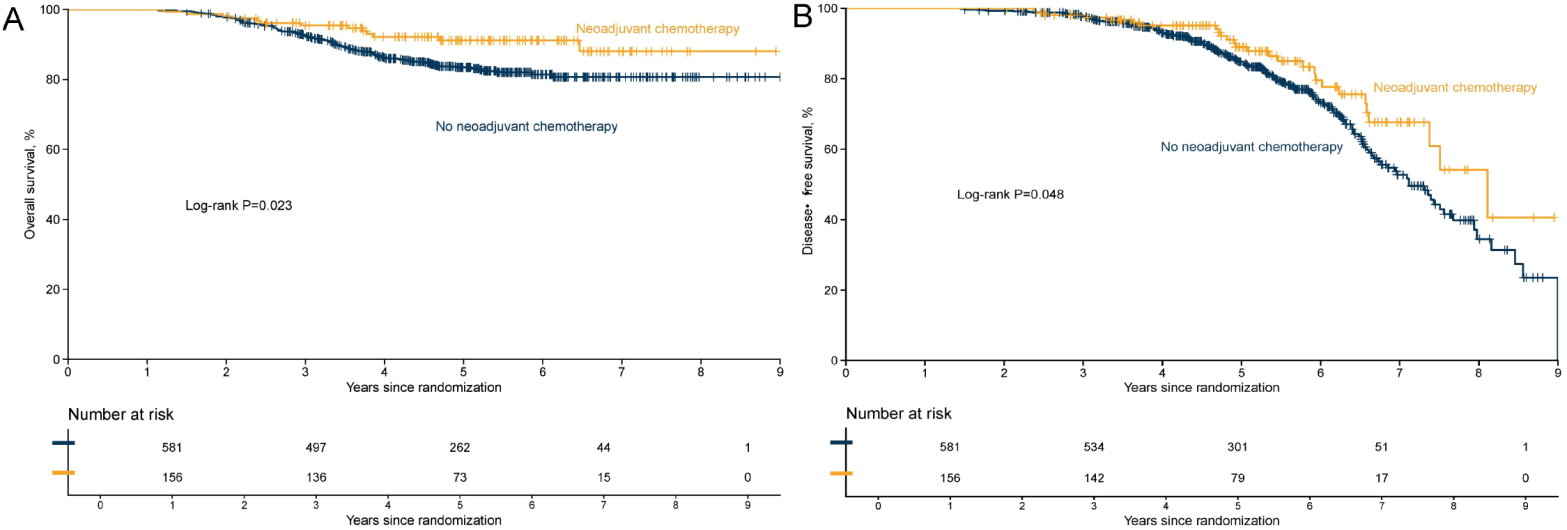
Kaplan–Meier curves of overall survival **(A)** and disease-free survival **(B)** in the Luminal B subgroup by neoadjuvant chemotherapy status.

**Figure 5:**
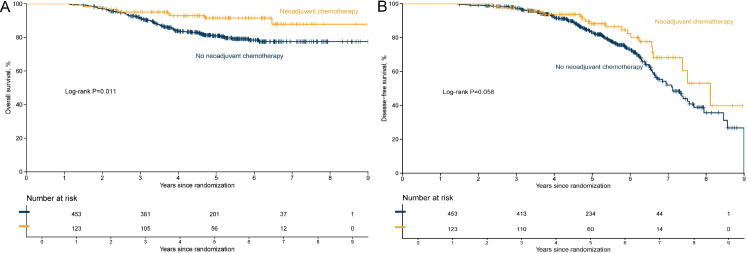
Kaplan–Meier curves of overall survival **(A)** and disease-free survival **(B)** in the high-risk Luminal B subgroup by neoadjuvant chemotherapy status.

**Figures 3**–**5** illustrate the Kaplan–Meier curves for OS and DFS in the overall population, Luminal B subgroup, and high-risk Luminal B subgroup, stratified by treatment.

In the overall study population, separation between the survival curves of the NACT and non-NACT groups was limited. For OS (**Figure 3A**), the NACT group showed a slightly higher survival probability, but the magnitude of difference was small. DFS (**Figure 3B**) showed a clearer separation trend, with the NACT group exhibiting a lower rate of invasive events during follow-up.

In the Luminal B subgroup, separation of the curves was more distinct than in the overall population. The OS curve (**Figure 4A**) showed that the NACT group maintained a higher survival probability throughout the follow-up period. For DFS (**Figure 4B**), the risk of recurrence or progression was lower in the NACT group, and the difference was more prominent compared with the overall population.

In the prespecified high-risk Luminal B subgroup (characterized by Luminal B biology plus high T/N stage and Ki-67), the survival curves showed an overall trend of separation. For OS (**Figure 5A**), the NACT group demonstrated higher survival probabilities starting from early follow-up. For DFS (**Figure 5B**), the NACT curve generally lay above the non-NACT curve, suggesting a lower risk of recurrence/progression, although the difference between the two groups did not reach statistical significance (log-rank test P > 0.05).

Overall, from the general population to the Luminal B subgroup, and further to the high-risk Luminal B subgroup, the survival advantage of the NACT group showed a progressively strengthening trend, suggesting that patients with high-risk biological and clinical features may derive more definitive relative benefit.

### Subgroup analysis of overall survival and disease-free survival

**Figure 6:**
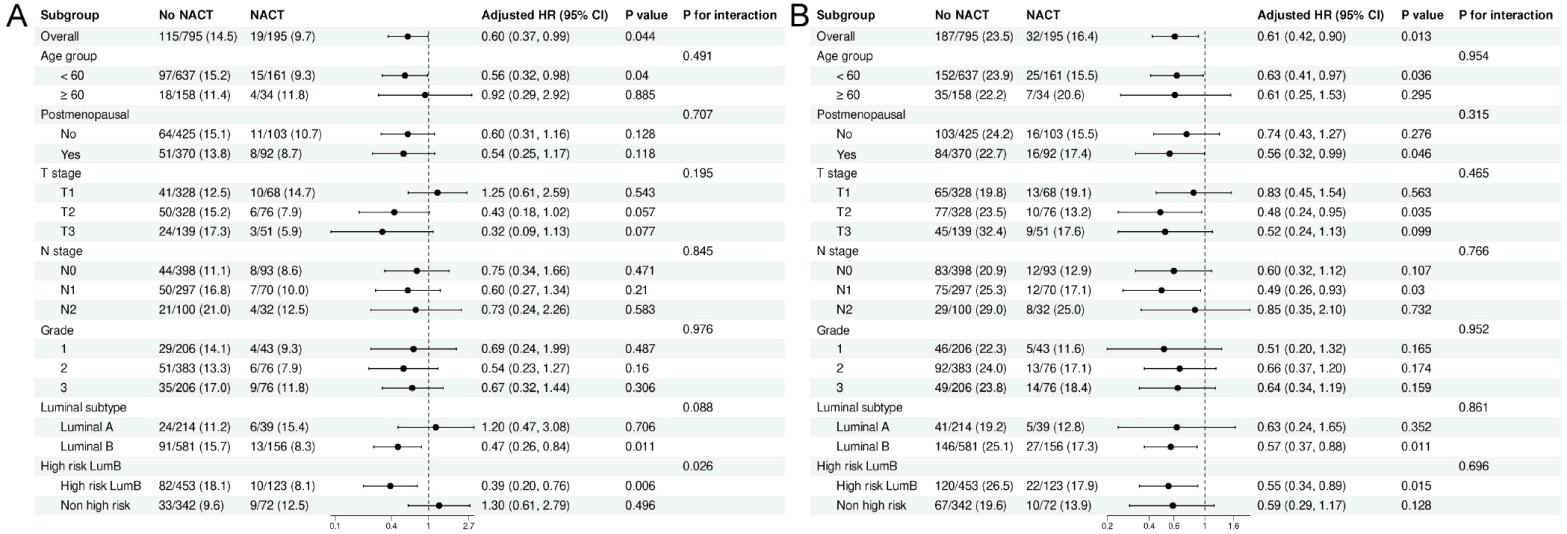
Subgroup analyses of overall survival **(A)** and disease-free survival **(B)** comparing neoadjuvant chemotherapy versus no neoadjuvant chemotherapy. Forest plots summarize subgroup analyses of overall survival (OS) **(A)** and disease-free survival (DFS) **(B)**. For each prespecified subgroup, the number of events and event rates (%) are shown for patients who received neoadjuvant chemotherapy (NACT) and those who did not. Adjusted hazard ratios (HRs) with corresponding 95% confidence intervals (CIs) were derived from multivariable Cox proportional hazards models including age, menopausal status, T stage, N stage, tumor size, grade, ER percentage, PR percentage, Ki-67 index, and luminal subtype. P values represent the significance of treatment effect within each subgroup, and “P for interaction” evaluates whether the treatment effect differed across subgroup levels. The vertical dashed line indicates HR = 1.0.

**Figure 6** displays the results of subgroup analyses for the effect of NACT on OS and DFS.

For OS (**Figure 6A**), in most clinical subgroups, the HR for NACT was < 1.0, indicating an overall trend favoring NACT, although the confidence intervals for many subgroups crossed 1. Notably, in the high-risk Luminal B subgroup, the interaction was statistically significant (P for interaction = 0.026). Within this subgroup, NACT significantly reduced the risk of death (adjusted HR = 0.39). In contrast, among non-high-risk Luminal B patients, NACT showed no OS benefit (adjusted HR = 1.30). This indicates significant heterogeneity in the relative treatment effect of NACT on OS across different Luminal B risk strata, with OS benefits primarily concentrated in the biologically and clinically high-risk Luminal B population.

For DFS (**Figure 6B**), HRs in most subgroups were < 1, but confidence intervals were generally wide, and no interaction tests reached statistical significance (all P > 0.05). Although the high-risk Luminal B subgroup also showed a numerical risk reduction, the difference did not reach statistical significance, suggesting no obvious heterogeneity in treatment effect for DFS across subgroups.

In summary, NACT demonstrated a significant relative benefit for OS in high-risk Luminal B patients, while for DFS, although the trend was consistent, no significant interaction effect was observed.

## Discussion

In this single-center, real-world study of 990 consecutive patients with HR-positive/HER2-negative (HR+/HER2−) early breast cancer, we systematically compared the impact of neoadjuvant chemotherapy (NACT) versus upfront surgery on long-term prognosis. Overall, despite the NACT group exhibiting significantly higher baseline risks—characterized by larger tumors, more T3 stage disease, higher histological grade, and elevated Ki-67 levels—multivariable Cox regression analysis revealed that NACT was independently associated with a lower risk of death (OS) and invasive disease-free survival events (DFS). In the overall population, the adjusted HRs were approximately 0.60 and 0.61, respectively. This benefit was even more pronounced in the Luminal B subgroup (adjusted HRs for OS and DFS were approximately 0.47 and 0.57, respectively). While survival analysis showed no significant difference in OS or DFS between the two treatment groups in the overall population (log-rank P > 0.05), significant differences in OS were observed within the Luminal B and high-risk Luminal B subgroups. Crucially, subgroup analyses demonstrated a statistically significant interaction between treatment modality and high-risk Luminal B status. This finding suggests that the long-term survival benefit of NACT is primarily concentrated in the high-risk Luminal B population, which is characterized by both high biological aggressiveness and significant clinical tumor burden.

The question of whether NACT improves long-term outcomes compared with upfront surgery (typically followed by adjuvant chemotherapy) in HR+/HER2− breast cancer remains contentious, with inconsistent findings across the literature. At the level of randomized evidence, an EBCTCG meta-analysis of individual patient data from 10 trials (n=4,756) (29) showed that shifting the same chemotherapy regimen from the postoperative (adjuvant) to the preoperative (neoadjuvant) setting generally resulted in equivalent distant recurrence and breast cancer mortality/overall survival rates. However, NACT was associated with a higher risk of local recurrence, suggesting that a simple “timing shift” does not automatically translate into a long-term survival advantage and that differences in local control strategies may influence outcomes. Real-world evidence is even more divided and susceptible to selection bias. For instance, a SEER database study (30) in T2N1M0 HR+/HER2− patients observed worse OS and breast cancer-specific survival (BCSS) with NACT compared with adjuvant chemotherapy (ACT) after propensity score matching. Notably, patients who did not achieve pCR had significantly worse outcomes than the ACT group (OS HR 1.58; BCSS HR 1.70), emphasizing that NACT might be detrimental due to “delayed definitive surgery plus failure to achieve deep response” in chemo-insensitive tumors. Conversely, another larger SEER study (19) in stage IIB–IIIC ER+/HER2− disease reported that NACT was generally associated with worse BCSS (multivariable HR 1.39) but highlighted a pivotal exception: in specific subgroups, particularly patients aged <40 years who achieved pCR, NACT was associated with better BCSS. This implies that the “benefit” is not evenly distributed across the HR+/HER2− population but is restricted to a minority of highly chemo-sensitive patients capable of achieving deep remission. Importantly, these studies from non-Asian populations provide a broader international context for interpreting our findings and support the view that the benefit of neoadjuvant chemotherapy in HR+/HER2− disease may be confined to selected biologically high-risk subgroups.

These conflicting observations likely stem from four factors: First, in real-world practice, NACT is often preferentially prescribed to higher-risk patients, and residual confounding may lead to the misinterpretation of “high-risk baseline” as “harm from NACT.” Second, analyzing HR+/HER2− as a monolithic entity obscures the biological differences between Luminal A-like and Luminal B-like tumors (e.g., high proliferation, low PR, high grade), thereby diluting the signal in subgroups that might truly benefit. Third, reliance on pCR as a core surrogate endpoint is problematic in HR+/HER2− disease due to its inherently low pCR rates (13–15), leading to structural conclusions of “no benefit” or “benefit seen only in the rare few with pCR.” Fourth, NACT alters the surgical approach, pathological assessment, and adjuvant treatment escalation pathways (and even local control strategies), creating variability in treatment chains across centers and eras. Our observation that the “benefit gradient” is concentrated in high-risk Luminal B patients, with a significant interaction for OS, provides an explanatory framework: NACT does not confer a net benefit to all HR+/HER2− patients but is more likely to manifest efficacy in populations combining “proliferation-driven Luminal B biology” with “higher tumor burden.” Conversely, in low-risk or chemo-insensitive HR+/HER2− cohorts, NACT is more likely to show no difference or even detriment, thereby resolving the apparent contradictions in previous studies.

From a mechanistic perspective, the concentration of the “benefit gradient” in high-risk Luminal B tumors is biologically plausible. It likely reflects the superposition of two risk axes: “proliferation-driven biology” and “higher tumor burden (T/N),” which determine the marginal benefit of chemotherapy. First, Luminal B-like tumors typically exhibit higher proliferative activity (elevated Ki-67, higher grade, or lower PR), consistent with a more active cell cycle and potentially higher endocrine resistance. This creates a window of sensitivity that cytotoxic chemotherapy is more likely to exploit compared with Luminal A-like tumors (31, 32). In our cohort, the NACT group’s higher Ki-67 levels and higher proportion of Luminal B tumors provide a baseline background consistent with this biological rationale. Furthermore, studies such as POETIC (33) have shown that Ki-67 levels and their early changes after perioperative endocrine therapy have clear prognostic stratification value, suggesting that the “proliferation phenotype” itself is a critical biological dimension of risk and treatment response in HR-positive tumors. Therefore, incorporating a higher Ki-67 threshold into the definition of “high-risk Luminal B” essentially structures the identification of a population—within the clinically accessible IHC framework—that is more likely to require, and benefit from, intensification.

Another finding with immediate clinical relevance is that clinical N stage remained the strongest and most stable adverse prognostic factor in both the overall population and the Luminal B subgroup. This aligns with established knowledge: in the HR+/HER2− context, even if the tumor retains some endocrine sensitivity, nodal burden represents a higher risk of micro metastasis and poorer long-term outcomes, constituting a core “clinical risk axis” in perioperative decision-making. Within this framework, it is not surprising that the benefit of NACT is concentrated in high-risk Luminal B patients. In our study, this subgroup aggregated higher tumor burden (higher T/N) with more active proliferative biology (higher Ki-67/grade). Theoretically, these patients have the greatest need for systemic therapy intensification and are most likely to derive marginal benefit from shifting systemic therapy to the neoadjuvant setting. In contrast, among non-high-risk Luminal B patients, NACT showed no OS benefit and even a trend toward harm (adjusted HR ~1.30). This phenomenon suggests that for HR+/HER2− patients with lower risk or more biologically indolent disease, NACT may represent a scenario of “limited benefit with significant toxicity and resource consumption.” Consequently, treatment decisions for these patients should be more cautious, favoring strategies centered on surgery plus endocrine therapy, or comparison with other perioperative escalation methods.

Our interpretation of the DFS results requires a degree of caution. Although the NACT group showed a consistent numerical advantage in 5-year DFS across the overall, Luminal B, and high-risk Luminal B populations, the interaction test for DFS in the high-risk Luminal B subgroup did not reach statistical significance. This may reflect multiple factors: First, DFS events are more subject to variability in follow-up intensity, sensitivity of recurrence detection, and subsequent therapies than OS, introducing greater uncertainty in real-world settings. Second, the sample size of the high-risk subgroup was smaller, leading to wider confidence intervals. Third, NACT may be more sensitive in reducing fatal recurrences or improving salvageability (impacting OS) than in preventing the “first invasive event” (impacting DFS). Therefore, the robust interpretation of our study is that NACT provides a clearer relative benefit signal for OS in high-risk Luminal B patients, while the DFS benefit, although directionally consistent, requires confirmation of its magnitude and mechanistic pathway in higher-powered studies. In addition to tumor biological characteristics and clinical tumor burden, alterations in host immune function may also contribute to disease progression and survival outcomes in breast cancer. Natural killer (NK) cells play an important role in tumor immune surveillance by recognizing and eliminating malignant cells. Previous studies have shown that breast cancer progression may be associated with impaired NK cell activity and altered metabolic characteristics of peripheral blood lymphocytes, suggesting a reduced capacity of the immune system to eliminate tumor cells. Such immune dysfunction may contribute to tumor progression and poorer clinical outcomes (34).

We must also acknowledge the limitations and alternative explanations of this study. The retrospective, non-randomized design inevitably introduces treatment selection bias and residual confounding. Although we adjusted for key variables such as age, menopausal status, T/N stage, tumor size, grade, ER/PR, Ki-67, and subtype in multivariable models, unmeasured factors such as comorbidities, performance status, chemotherapy dose intensity/adherence, socioeconomic factors, and adjuvant treatment escalation pathways could not be fully accounted for. Additionally, Ki-67 is subject to inter-laboratory and inter-observer variability (35, 36). While single-center testing favors consistency, there remains a risk of misclassification when using Ki-67 ≥30% to define the high-risk subgroup. Furthermore, pathological complete response (pCR) and residual cancer burden (RCB) data were not systematically available in the institutional database, and therefore were not included in the present analysis. As a result, we were unable to evaluate the relationship between pCR and long-term survival outcomes in this cohort. Consequently, we cannot fully distinguish whether the survival improvement stems from the downstaging effect of chemotherapy itself or from the comprehensive impact of the “perioperative treatment chain.” Future replication of the OS interaction signal in multi-center prospective cohorts, utilizing stricter confounding control methods (e.g., propensity score matching/weighting) and integrating response depth (pCR/RCB), residual disease information, and postoperative escalation into a single causal pathway analysis, will help clarify “who benefits, why they benefit, and how the treatment chain delivers that benefit.”.

## Conclusion

In conclusion, this study supports a more actionable clinical message: In HR+/HER2− early breast cancer, the consideration of NACT should not be driven solely by the broad “HR+/HER2−” label. Instead, clinicians should integrate Luminal subtyping with T/N burden and proliferation phenotypes (such as Ki-67) to identify the high-risk Luminal B population—characterized by both biological and clinical high-risk features—as the priority target for potential long-term survival benefits from NACT. For these patients, NACT has the potential to evolve from a “tool for surgical feasibility” to an “active option for OS improvement.” Conversely, for Luminal A or non-high-risk Luminal B patients, the more rational path likely remains centered on upfront surgery and endocrine therapy, with decisions regarding chemotherapy intensification or other perioperative strategies made cautiously based on composite risk assessment.

## Data Availability

The original contributions presented in the study are included in the article/supplementary material. Further inquiries can be directed to the corresponding author.
